# Antioxidant Properties of Pecan Shell Bioactive Components of Different Cultivars and Extraction Methods

**DOI:** 10.3390/foods10040713

**Published:** 2021-03-27

**Authors:** Cameron Cason, Veerachandra K. Yemmireddy, Juan Moreira, Achyut Adhikari

**Affiliations:** 1 School of Nutrition and Food Sciences, Louisiana State University AgCenter, 261 Knapp Hall, Baton Rouge, LA 70803, USA; Cameron.cason@wellcana.com (C.C.); jmoreiracalix1@lsu.edu (J.M.); 2 School of Earth, Environmental, and Marine Sciences, University of Texas Rio Grande Valley, Edinburg, TX 78539, USA; krantiyemmireddy@gmail.com

**Keywords:** pecan, antioxidant, extraction, cultivar

## Abstract

Pecan shells are a rich source of various bioactive compounds with potential antioxidant and antimicrobial properties. This study investigated the effect of pecan variety and method extraction on the antioxidant property of shell extracts. Twenty different varieties of pecan shells were subjected to either aqueous or ethanolic extraction and were examined for total phenolics and antiradical activity. The phenolic content and antiradical activity of shell extracts were significantly (*p* < 0.05) varied with different pecan cultivars. The total phenolic content of ethanol extracts ranged from 304.2 (Caddo) to 153.54 (Cherokee) mg GAE g^−1^ of dry extract and was significantly greater (*p <* 0.05) than those obtained by aqueous extraction. The antiradical activity of ethanol extracts ranged from 840.6 (Maramec) to 526.74 (Caper Fear) mg TE g^−1^, while aqueous extracts ranged from 934.9 (Curtis) to 468.3 (Elliot) mg TE g^−1^. Chemical profiling of the crude and acid hydrolyzed extracts was performed by reverse phase high performance liquid chromatography and flow injection electrospray ionization mass spectrometry. Lignin degradation products such as lignols, dilignols, trilignols, and oligolignols were found to be the major components of tested extracts. Phenolic content and antiradical activity of pecan shell extracts are significantly varied with cultivars and methods of extraction.

## 1. Introduction

The natural foods sector has undergone a significant growth over the past decade [1]. This is partly due to consumers’ consciousness of potential health risks associated with synthetic ingredients. In response, demands are shifting away from food products preserved by conventional chemical or physical methods, in favor of “more natural” or “organic” products [2]. Pecans are one of the most popular edible nuts in the USA. Over 270 million pounds are produced annually in the United States. Following harvest, over 90% of pecan nuts are processed to remove the outer shell layer, and only the kernel is sold for consumption [3]. The shell by-product constitutes approximately 50% of the harvested mass, which corresponds to nearly 6 million pounds of by-product per annum that is underutilized [4]. Recently plant bioactive compounds have gained attention for their functional properties. Several studies have demonstrated that pecan shells are a rich source of phenolic compounds, ranging from phenolic acids to flavan-3-ols and anthocyanins [5,6,7,8]. In certain cases, the total phenolic content of pecan shells has been found to be 60% higher than that of kernels. Likewise, the total flavonoid content of pecan shells has been determined to be five times higher than that of kernels [9]. These compounds are known to have antioxidant and antimicrobial properties. These antimicrobial properties have effectively reduced the growth of *L. monocytogenes* on catfish fillets and fresh-cut cantaloupes [10]. Thus, pecan shells have the potential to be used as an alternative source of natural antioxidants in various food applications.

Many factors, such as growing region [7,11], cultivar [5,12], cultivation method [13], and harvest year [14] have been shown to significantly affect the bioactive profile and antioxidant activity of pecan components. However, there exists a knowledge gap as regards an extensive comparison of the effect of cultivars across a large population while controlling the harvest year, growing region, and cultivation method on antioxidant properties. Extensive breeding efforts in the United States between 1960 to 1980 have led to the development of over 500 pecan cultivars. Cultivars commonly called “improved varieties” are bred to be more resistant to environmental stresses and produce nuts with thin shell walls and kernels that are high in lipid content and may resist lipid oxidation over long storage times [4]. Stress adaptation is an action of secondary bioactive components produced by the plant. It has been suggested that the shell’s phenolics and antioxidant activity are higher in cultivars with kernels containing high amounts of lipids [8,14]. Geographical location may affect the phenolic profile of the kernel and nutshells [7], with significant differences in phenolic content between pecans produced by different cultivation methods [13].

The antioxidant activity of extractable shell bioactive components of pecan cultivars produced in the southern region of the United States has not been well studied. Furthermore, there is a lack of comparative studies on extraction methods to obtain extracts with the highest antioxidant activity. Comparative studies on extraction methods are limited and typically only compare a single cultivar. There are some analytical difficulties such as interfering components, inefficient separation, and the use of destructive techniques, that have limited shell characterization studies. This warrants the study on the effect of the cultivar and extraction method on shell bioactive components of pecans cultivated in the southern region of United States. Thus, the main objectives of this study are: (i) To evaluate the effect of the cultivar and method of extraction on the phenolic content and antiradical activity of shell extracts of pecans grown in southern United States; and (ii) To characterize the bioactive components in different pecan shell extracts.

## 2. Materials and Methods

### 2.1. Chemicals and Reagents

All reagents and standards that were used in chemical assays were American Chemical Society (ACS) grade. Folin–Ciocalteu phenol reagent, DPPH (2,2-Diphenyl-1-picrylhydrazyl), gallic acid (3,4,5-Trihydroxybenzoic acid), trolox (6-Hydroxy-2,5,7,8-tetramethylchroman-2-carboxylic acid), sodium carbonate monohydrate, ACS grade solvents hexane, methanol, and hydrochloric acid, as well as HPLC grade acetic acid and acetonitrile, were purchased from VWR International (Radnor, PA, USA), while ethanol (95% *v/v*) was supplied by Louisiana State University Stores (Baton Rouge, LA, USA).

### 2.2. Pecan Cultivars

Various cultivars of in-shell pecans (*Carya illinoinensis* (Wangenh) C. Koch) were obtained from Louisiana State University AgCenter’s Pecan Research and Extension Station (Shreveport, LA, USA). The pecans used in this study were harvested in September through November 2017, after having received all the same fertilization and pesticide applications. The pecans were obtained from three different orchards and were sampled from trees of different ages. Cape Fear, Creek, Gloria Grande, Jackson, Maramec, and Melrose cultivars were grown in the *Northwest* orchard, established in 1981. Cherokee, Curtis, Kiowa, Moreland, Point Coupee, Schley, Success, and Summer were harvested from the *Pathology* orchard, established in 1988. The youngest orchard sampled was the *Demo* orchard, having provided nuts from trees planted in 2005.

### 2.3. Sample Preparation

Upon receiving the pecans at the School of Nutrition and Food Sciences, Louisiana State University, Baton Rouge, the pecans were stored at a refrigerated temperature (4 °C) until further use in the experiments. Pecans from 20 cultivars (Table 1) were removed from the refrigerated storage and individually cracked using a nutcracker, and the shells were then separated. This process was not done in triplicates due to the amount for each pecan cultivar provided being limited. Later, the shells were crushed to a smaller size before being dried in a convection oven (Model 1370 GM, SHEL LAB, VWR) for 8 h at 40 °C. Dried nut shells were ground into a powder using a food processor (MB-1001C, Magic Bullet) and stored in 250 mL amber colored glass bottles at −20 °C for future use.

NSP (nut shell powder) from each cultivar was transferred from cold storage and allowed to equilibrate to 23 °C. Solid-liquid extraction was used to remove lipids from pecan shells. NSP from each cultivar (8 g) was individually weighed and placed in a new 250 mL amber colored bottle. A volume of 160 mL of hexane (1:20 *w*/*v*) was added to each bottle and then thoroughly mixed at 160 rpm using an Incubator Shaker (C25KC, New Brunswick Scientific, Edison, NJ, USA) for 45 min at 22 °C. Hexane was then slowly filtered from the pecan shell residue using a Buchner funnel equipped with a filter paper (Whatman^®^ No. 1) under vacuum. This process was repeated twice, and the defatted pecan nut shell powder cakes were placed inside a chemical hood for 4 h in the absence of light to allow the residual hexane solvent to evaporate. The defatted samples were stored in 250 mL amber colored bottles in the absence of light at −20 °C.

### 2.4. Bioactive Extraction

Solid-liquid extraction using either distilled water or ethanol (95% *v*/*v*) was used to extract bioactive compounds from the defatted NSP. Prior to extraction, the defatted NSP was removed from the freezer (−20 °C) and allowed to equilibrate to 23 °C. To perform aqueous extractions, a 2 g aliquot of defatted NSP from each cultivar was weighed (Mettler Toledo XS204, Greifensee, Switzerland) and placed into individual 250 mL amber bottles. Aqueous infusions (20 g/L) were prepared by pouring 160 mL distilled water at 98 °C into each 250 mL amber bottle containing defatted NSP. The bottles were quickly capped and placed in a Buchi™ 461 hot-water bath (98 °C for 30 min), with mixing at every 5 min. Following extraction, aqueous infusions were removed from the hot water bath and allowed to cool for 10 min. The extracts were then filtered under vacuum using a Buchner funnel equipped with a filter paper (Whatman^®^ No. 1, Maidstone, UK). The extracts were collected in individual 250 mL amber bottles, and the pecan shell residue was re-extracted following the same procedure. The extracts from the first and second aqueous fractions were combined and stored in a freezer at −80 °C. Ethanolic extracts were prepared by mixing defatted NSP with ethanol (20 g/L) in 250 mL amber colored bottles and were constantly mixed at 160 rpm using the incubator shaker for 60 min at 22 °C. Then, the extracts were filtered as previously described and stored at −80 °C. The extracts were concentrated to a powder by lyophilization. Prior to chemical analysis, aliquots of lyophilized extracts were diluted in methanol (0.2 mg/mL), vortexed, and filtered (0.45 µm).

### 2.5. Determination of Phenolic Compound

The total phenolic content of aqueous and ethanolic extracts of pecan shell was estimated by the Folin–Ciocalteu colorimetric assay using a microtiter plate according to Singleton, Orthofer, and Lamuela-Raventos 1999 [15]. In a 96-well microplate, 30 µL aliquots of each freeze-dried diluted extract were mixed with 150 µL of Folin–Ciocalteu reagent (1:10, *v*/*v* in distilled water). After 5 min, the reaction was neutralized with 120 µL sodium carbonate (75 g/L) and then incubated at 22 °C for 90 min in the dark. The absorbance of the resulting reactions was measured via a microplate reader (Bio-Rad^®^ Benchmark Plus, Hercules, California) at 765 nm. A gallic acid standard curve (300, 250, 200, 150, 100, 75, 50, 25 µg/mL) was generated as a reference, and the data were expressed as mg gallic acid equivalents per gram of freeze dried extract (mg GAE g^−1^). The analyses were carried out in duplicates, with three replications in each.

### 2.6. Evaluation of Antiradical Activity

The evaluation of the antiradical potential of the shell extracts was conducted using a DPPH (2, 20-azinobis-(3-ethyl-benzothiazoline-6-sulfonic acid)) free radical assay as described by Brand-William, Cuvelier, and Berset (1994), with some modifications [16]. In a microplate, a 10 µL aliquot of the diluted extracts was mixed with 200 µL of DPPH (0.01 M DPPH in methanol). The plate was covered and incubated in the dark at 22 °C for 30 min. A microplate reader (Eppendorf™ AF2200, Hamburg, Germany) was then used to measure the initial and final absorbance at 540 nm. The radical scavenging activity was calculated according to the following equation:

$$\mathrm{Radical}\;\mathrm{scavenging}\;\mathrm{effect}\;\left(\%\right)=\frac{\left(A_{540}\;0\;\min-A_{540\;}30\;\min\right)}{A_{540}}\times 100$$

A Trolox standard curve (500, 250, 200, 100, 50, 25, 10 µg/mL) was generated to quantify the antiradical activity of the extracts. Results were expressed as mg Trolox equivalents per gram of freeze dried extract (mg TE g^−1^). The analyses were carried out in duplicates, with three replications each.

### 2.7. Reverse Phase High Performance Liquid Chromatography (RP-HPLC)

Following the pre-screening of crude extracts from all 20 cultivars for total phenolics and antiradical activity, aqueous and ethanolic extracts from one high (Caddo) and one low (Nacono) performing cultivar were selected for chemical profiling. RP-HPLC with UV/VIS absorbance detection was used to characterize crude and acid hydrolyzed extracts. Acid hydrolysis was performed to free glycosidic bound phenolic compounds. The crude extracts were weighed and placed in 250 mL amber bottles containing acidified methanol (1% HCl *v*/*v*) for 24 h under constant shaking (160 rpm) at 23 °C. The resulting extracts were centrifuged at 6500× *g* for 6 min, and the resulting supernatant was dried using an evaporator (Labconco™ 7812013 Centrivap, Kansas City, MI, USA) at 70 °C. Extracts were diluted in methanol (25 mg/mL) and then centrifuged at 12,000× *g* for 10 min to remove insoluble material and then transferred to a 1.5 mL vial for analysis. Chromatographic separations of extracts were performed using a HPLC system (Model 2690, Waters Alliance™, Milford, MA, USA) equipped with a 996-photodiode array detector. Bioactive compounds were separated using 4.6 mm × 250 mm C18 column. 50 µL of extract was eluted in a bi-solvent mobile phase composed of aqueous acetic acid (10% *v*/*v*) (solvent A) and acetonitrile (solvent B) for a total run time of 94 min. Prior to sample injection, the column was equilibrated with 100% solvent A. Upon injection, the samples were eluted at a flow rate of 0.8 mL min^−1^ with the following gradient: A 100% for 0–50 min, A 70% and B 30% 50–70 min, A 50% and B 50% 70–80 min, A 20% and B 80% 80–85 min, B 100% 85–90 min, and A 100% 90–94 min.

### 2.8. Flow Injection Analysis Mass Spectrometry

Flow injection analysis mass spectrometry (FLA-ESI-MS) using an Advion expression^L^ Compact Mass Spectrometer (CMS) was performed on acid hydrolyzed Nacono ethanolic extracts to confirm the potential compounds identified using RP-HPLC-DAD. An ethanolic extract was selected for this study because it was found to be more efficient at extracting bioactive components of higher antiradical activity compared to distilled water. A 5 µL volume of extract was manually injected and ionized with either electrospray ionization (ESI) or a typical fragmentation setting with acetonitrile (75% *v*/*v*) as a mobile phase. Positive and negative ions from 50–1200 amu were recorded in the mass spectrums. Background noise was collected and subtracted from the total ion count chromatograms.

### 2.9. Statistical Model

The effect of the extraction method was evaluated under the assumptions that the total phenolic content (TPC) or the free-radical scavenging activity of the aqueous and ethanolic pecan shell extracts from the corresponding cultivars were equal (H_0_: *μ*_aqueous_ = *μ*_ethanolic_). The claim that either the TP or DPPH of the ethanolic and aqueous extracts from the corresponding pecan cultivars were different was tested using a two-sided paired t-test (*p* ≤ 0.05) on replication means (Ha: *μ*_aqueous_ ≠ *μ*_ethanolic_). This t-test is appropriate for our data set because it allows us to determine if a difference exists between two values that correspond to a common group. In our analysis we are comparing either the total phenolics or the antiradical activity of the extractions obtained by two different extraction methods on a common cultivar. The effect of the cultivar on the phenolic content and the antiradical activity of the ethanol and aqueous extracts was evaluated using a two separate one-way analysis of variance (ANOVA), with a post hoc Tukey (HSD) test (*p* ≤ 0.05).

## 3. Results and Discussion

### 3.1. Effect of Extraction Method on Total Phenolic and Antiradical Activity

**Figure 1:**
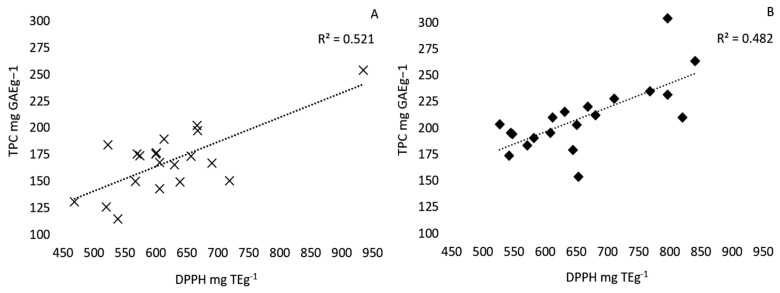
Correlation between the total phenolic content and antiradical activity of 20 pecan cultivar extracts. Positive linear correlation between the total phenolic content (TPC) reported as mg GAE g^−1^ (gallic acid equivalents per gram of dry extract) measured using the Folin–Ciocalteu assay and antiradical activity measured by the DPPH method reported in mg TE g^−1^ (Trolox equivalents per gram dry extract) of 20 pecan cultivars extracted by distilled water (20 g/L, 30 min, 22 °C) (**A**) or ethanol (20 g/L, 1 h, 22 °C) (**B**) solid-liquid extraction.

The yield of pecan shell extracts from 20 different cultivars obtained by either aqueous or ethanol solid-liquid extraction is reported in Table 1. Large differences were observed in the yield of pecan shell extracts from 20 different cultivars. The yield of ethanolic extracts ranged from 240 (Pawnee) to 3 (Sumner) mg dry extract/g defatted shell powder, while aqueous extracts ranged from 490 (Caddo) to 89 (Jakson) mg dry extract/g defatted shell powder (Table 1). Crude extracts subject to different extraction methods yielded different total phenolic contents (TPC), measured by a Folin–Ciocalteu assay (Table 2). The TPC of ethanolic extracts ranged from 304.18 to 153.54 mg GAE g^−1^ of dry extract with an average of 210.02 ± 7.3 mg GAE g^−1^ and were significantly greater (*p <* 0.05) than those obtained by aqueous extraction, which ranged from 253.75 to 114.63, with an average of 168.38 ± 6.8 mg GAE g^−1^ of dry extract. However, the method of extraction did not significantly affect the free-radical scavenging activity measured by the DPPH assay (Table 2). These results are similar to those by Kureck et al. (2018), who obtained aqueous extracts and ethanolic extracts from the Barton variety with TPC yields of 186.02 ± 2.31 mg GAE g^−1^ and 275.24 ± 41.88 mg GAE g^−1^, respectively [17]. The free-radical scavenging activity of the ethanolic extracts ranged from 820.39 to 526.74 and averaged 659.70 ± 21 mg TE g^−1^, while the aqueous extracts ranged from 934.95 to 468.34, with an average of 619.42 ± 22 mg TE g^−1^. Kureck et al. (2018) determined that the DPPH of the Barton variety pecan shell ethanolic extracts was significantly larger than the aqueous extracts, which is in accordance with our findings [17]. A positive linear correlation between phenolic content and antiradical activity was observed for the aqueous (R^2^ = 0.52) and ethanolic extracts (R^2^ = 0.48) (Figure 1). Pecan shell aqueous infusions were found to have a much stronger linear relationship (R^2^ = 0.99) when extracts were not dried prior to analysis [12]. In disagreement with this study, Prado et al. (2009) reported that the TPC (181.49 ± 6.97 mg GAE g^−1^) and antiradical activity of aqueous extracts (DPPH 612.24 ± 26.73 mg TE g^−1^, ABTS 1809.01 ± 27.18 mg Teg^−1^) was significantly greater than those of ethanol extracts (167.85 ± 3.89 mg GAE g^−1^, DPPH 524.77 ± 40.72 mg Teg^−1^, ABTS 1562.51 ± 33.15 mg Teg^−1^) [12]. High gallic acid and epigallocatechin gallate content was strongly associated with high antioxidant activity measured by the DPPH assay. Interestingly, the condensed tannin content of the ethanol extracts was 11 times greater than that of the aqueous extracts [6]. Our data is in agreement with Prado et al. (2014) and Villareal-Lozoya et al. (2007) [5,6]. They reported that acetone:water (70:30) extracts from defatted shells contained 10–23 times greater condensed tannin content compared to aqueous extracts. This indicates that the extraction efficiency of condensed tannins is increased when an organic solvent is used.

The antioxidant properties of pecan nuts have been attributed to phenolic compounds. The Folin–Ciocalteu colorimetric assay is widely used to estimate the total phenolic content (TPC) of plant extracts. However, this method is not specific for phenolic compounds and is sensitive to other reducing agents. The DPPH free-radical scavenging assay is used to measure the plant extracts’ ability to retard free-radical initiated oxidation. Together, these assays are an indicator of the total relative antioxidant potential. Sánchez-Rangel et al. (2013) and Chun and Kim (2014) showed that monomeric phenolics were less reactive to the Folin–Ciocalteu reagent compared to their multimeric derivates [18,19]. It was suggested that higher degrees of flavonoid polymerization predict an increase in antiradical activity measured by a DPPH assay [20].

### 3.2. Effect of Cultivar on the Phenolic Content and Free-Radical Scavenging Activity

The pecan cultivar significantly (*p* ≤ 0.05) affected both the TPC and the free-radical scavenging activity of the aqueous and ethanolic extracts. When considering the aqueous extracts, the tested cultivars ranked from highest to lowest TPC are as follows: Curtis ≥ Pawnee ≥ Schley ≥ Point-Coupee ≈ Maramec ≥ Caddo ≈ Oconee ≈ Sumner ≈ Nacono ≈ Kiowa ≈ Success ≈ Desirable ≥ Cherokee ≈ Moreland ≥ Gloria Grande ≈ Creek ≥ Cape Fear ≥ Elliot ≥ Melrose > Jackson (Table 2). The free-radical scavenging activity of the aqueous extracts followed the trend: Curtis ≥ Moreland ≈ Desirable ≈ Schley ≈ Pawnee ≥ Kiowa ≈ Creek ≈ Cherokee ≈ Point Coupee ≈ Cape Fear ≈ Success ≈ Caddo ≈ Oconee ≈ Nacono ≈ Sumner ≈ Gloria Grande ≈ Jackson ≈ Maramec ≈ Melrose ≈ Elliot. Prado et al. (2009) reported that aqueous shell extracts from a mixture of Barton (approximately 50%), Shashone, Shawnee, Choctaw, and Cape Fear were lower in phenolic content (138 ± 26 mg GAE g^−1^) and antioxidant activity (572 ± 102 mg TEACg^−1^) compared to the respective averages for aqueous extracts in this study [12]. In this study, methanol soluble components of aqueous extracts were quantified in methanol for chemical assays, while Prado et al. (2009) assayed extracts in an aqueous solution [12]. The TPC of ethanolic extracts followed the trend: Point-Coupee > Maramec ≥ Elliot ≈ Gloria Grande ≈ Jackson ≈ Melrose ≈ Moreland ≈ Caddo ≈ Desirable ≥ Curtis ≈ Cape Fear ≈ Creek ≈ Pawnee ≈ Sumner ≈ Schley ≈ Kiowa ≈ Oconee ≈ Nacono ≈ Success > Cherokee (Table 2). The antiradical activity of the extracts obtained by ethanol extraction followed the trend: Maramec ≥ Curtis ≥ Point Coupee ≥ Elliot ≥ Gloria Grande ≈ Jackson ≈ Caddo ≈ Melrose ≈ Cherokee ≈ Creek ≈ Moreland ≥ Desirable ≈ Pawnee ≥ Kiowa ≈ Nacono ≈ Oconee ≥ Schley ≥ Sumner ≈ Success ≈Cape Fear (Table 2).

Villareal-Lozoya et al. (2007) showed that the cultivar significantly affected (*p* < 0.05) the total phenolic content (TPC) assayed by a Folin–Ciocalteu assay and the antiradical capacity (DPPH assay) of dried shell extracts obtained using acetone:water (70:30 *v*/*v*) as a solvent from six different cultivars that were harvested from the same orchard in 2007 [5]. Kanza (TPC 633 ± 29 mg CAE g^−1^, DPPH 675 ± 18 mg TE g^−1^), followed by Pawnee (TPC 537 ± 10 mg CAE g^−1^, DPPH 582 ± 29 mg TE g^−1^), had the greatest phenolic content and antiradical activity. Other cultivars were also investigated, including Shawnee (TPC 537 ± 10 mg CAE g^−1^, DPPH 444 ± 3 mg TE g^−1^), Nacono (TPC 451 ± 6 mg CAE g^−1^, DPPH 442 ± 7 mg TE g^−1^), Desirable (TPC 378 ± 17 mg CAE g^−1^, DPPH 482 ± 30 mg TE g^−1^), and Kiowa (TPC 344 ± 10 mg CAE g^−1^, DPPH 331 ± 11). In comparison to Villareal-Lozoya et al. (2007), Pawnee was found to be significantly greater in phenolic content compared to the Nacono, Desirable, and Kiowa cultivars in extracts obtained with water and acetone:water (70:30 *v*/*v*) as extraction solvents [5]. Furthermore, Nacono extracts had a higher phenolic content compared to Desirable and Kiowa cultivars when these solvents were used. It is concluded that the phenolic and antiradical properties of pecan shell components are dependent on numerous factors in combination. Pecans cultivated in southern United States were found to be rich in antioxidant components. Ethanol was found to be better than distilled water as a solvent to extract phenolics from pecan shells. The antioxidant activity of the extracts obtained through distilled water or ethanol extraction was found to be highly dependent on the type of cultivar.

### 3.3. Bioactive Profile by RP-HPLC

**Figure 2:**
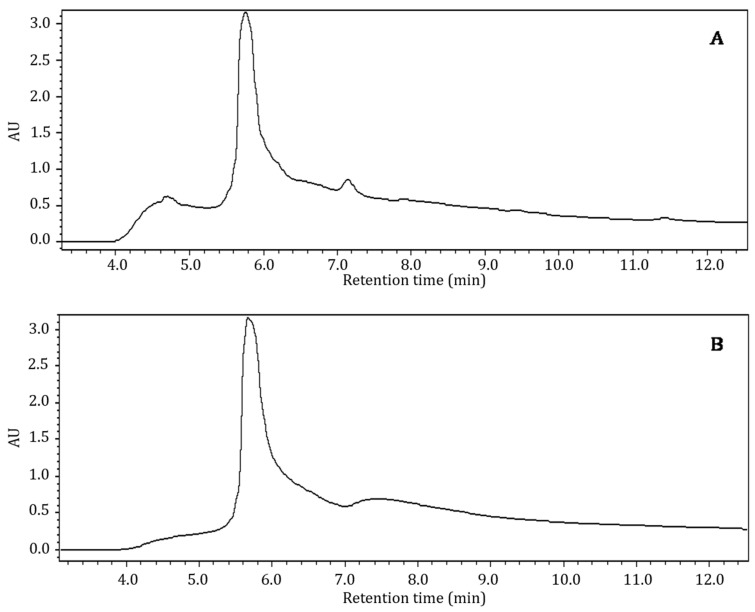
Bioactive profile of crude pecan shell Nacono extracts by reverse phase high performance liquid chromatography (RP-HPLC). Hyphenated chromatograms (3.5–12.2 min) by HPLC of crude pecan shell Nacono extracts. A 50 µL volume of methanol soluble components was injected into a c18 column, eluted with a binary mobile phase, and detected with a UV/VIS diode array absorbance detector. The chromatograms represent detection at the 280 nm wavelength channel. Pecan shells were subjected to solid-liquid extraction (20 g^−L^) with distilled water at 98 °C for 1 h (**A**) or ethanol at 22 °C for 1 h (**B**) under constant mixing.

Bioactive components in crude aqueous and ethanol extracts of Nacono and Caddo cultivars were analyzed by reverse phase HPLC with UV/VIS detection using a photodiode array detector. Retention times and absorption wavelengths of eluted components were compared to phenolic standards analyzed under similar conditions to presumably characterize the extracts. Methanol soluble components of crude shell extracts were eluted from the separatory column between 5.7 and 14.4 min (aqueous) and between 5.7 and 13.3 min (ethanol), in unresolved peaks with absorption bands between 280 and 460 nm (Figure 2). The most abundant peak in either extract eluted at approximately 5.7 min, with a peak area of 1.20 × 10^8^ and 1.28 × 10^8^ in the aqueous and ethanol extracts, respectively. However, components comparable to free phenolic standards were not resolved in the broad-shouldered peak of the crude extract chromatograms. Absorption in the ultraviolet and visible regions indicates a degree of aromaticity or conjugated double bonds. Specifically, absorption bands at 280 nm are associated with phenolic compounds and some amino acid structures, namely tyrosine and tryptophan. The component that gives the extracts a red hue is likely responsible for the absorption at 460 nm. Prado et al. (2013) reported that aqueous soluble shell components could be quantified by measuring absorbance at 420 nm [14]. Furthermore, the authors determined through principal component analysis that a deeper red color was associated with increased antioxidant activity and quantity of phenolics, protein, and fiber of aqueous shell extracts. Other studies have reported similar analytical challenges when characterizing phenolic components in crude extracts from pecan shell and kernel by RP-HPLC [5]. Many studies have suggested that pecan shell phenolics are primarily in oligomeric or bound forms as condensed or hydrolysable tannins or as glycosides. The use of extraction or analytical preparatory steps alters the native state of the compounds, often resulting in the loss of important structural information. Furthermore, many of the structures elucidated using these techniques may be a product of the analytical methods used to extract and analyze the components of interest. Hydrolysable tannins yield gallic and ellagic acid under weak acidic or basic conditions. The oxidative cleavage of condensed tannins (proanthocyanidans) with acid yields anthocyanidin pigments and phlobaphenes associated with a red color.

**Figure 3:**
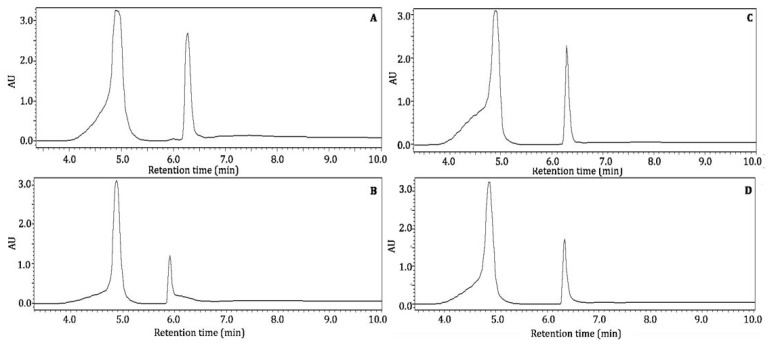
Bioactive profile of acidified hydrolyzed crude pecan shell Nacono extracts by RP-HPLC. Hyphenated chromatograms from 3.5 to 10.0 min of acidified methanol (1% HCl *v*/*v*) soluble components of crude extracts analyzed by reversed phase HPLC with detection at 280 nm, as described in Figure 2. Caddo aqueous extracts are represented by the letter (**A**), and Caddo ethanol extracts are labeled (**B**). The letter (**C**) corresponds to Nacono aqueous extracts; thus, letter (**D**) represents Nacono ethanol extracts.

In the present study, Nacono and Caddo crude extracts were extracted with acidified methanol (1% HCl *v*/*v*) to free, polymeric, or bound form phenolics. The soluble components were analyzed by RP-HPPLC-DAD with the same method used to analyze their crude constituents. Acid hydrolysis removed the interfering components in the chromatograms, resulting in the detection of two prominent and fully resolved peaks for all extracts (Figure 3).

The treatment of lignocellulose with diluted acid in a polar solvent cleaves ester and ether linkages to produce free monomeric phenols [21]. Furthermore, cleaved ester and ether bonds can reassociate into more complex polymeric structures. These modifications limit the reproducibility of pecan shell characterization studies [22]. Potential compounds of major methanol soluble bioactive components were elucidated by reverse phase HPLC in crude and acid hydrolyzed pecan shell extracts (Figure 3). A peak at 4.9 min with a maximum absorption wavelength of 280 nm was common in all extracts but was most abundant in aqueous extracts. This peak closely resembled that of gallic acid, with Rt 5.0 min and max absorption at 272 nm. The second major component eluted at Rt 6.3 with maximum absorption at 280 nm, which was not consistent with phenolic standards. It is hypothesized that this peak is a phenolic product derived from acidified methanol extraction. The quantification of these peaks was not attempted, but retention times and relative abundance are reported in Table 2. Rosa et al. (2011) identified only gallic and ellagic acid in acid-hydrolyzed acetonic extracts from pecan nutshell [11]. In another study, Rosa et al. (2014) showed that acetone:water (70:30 *v*/*v*) soluble epicatechin components in pecan shell are hydrolyzed to gallic and ellagic acid under acid conditions [7]. HPLC data provided little analytical information to conclusively characterize shell bioactive components. Thus, more powerful analytical methods were employed, as discussed below.

### 3.4. Bioactive Characterization by Flow Injection Electrospray Ionization Mass Spectrometry (FIA-ESI-MS)

**Figure 4:**
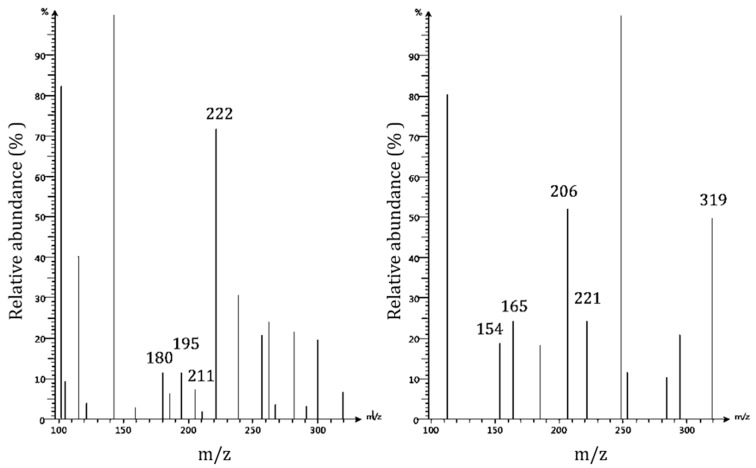
Bioactive characterization of acid hydrolyzed ethanolic extracts of Nacono cultivar by FIA-ESI-MS. Mass spectrums by flow injection-electrospray ionization-mass spectrometry (FI-ESI-MS) of acid hydrolyzed (1% HCl *v*/*v* in methanol) ethanolic extracts of Nacono cultivar with ion mode switching every second. Protonated ions (Left): Coniferyl alcohol (179 u), phenolic 8-end G(β-O-4′)G dilignol (*m*/*z* 195), Sinapyl alcohol S-lignol (210 u), and aliphatic 4-end of G(β-5′)G dilignol (221 u). Deprotonated ions (Right): Vanillyl alcohol (154 u), guaiacylpropane (166 u), phenolic 8-end of G(β-β′)G dilignol (*m*/*z* 206), aliphatic 4-end of G(β-5′)G dilignol, and guaiacylglycerol-β-guaiacylether (320 u).

Mass spectrometry is an analytical technique used to determine the molecular masses of analytes by creating ions and separating them by their mass to charge ratio. This technique can provide structural information with a molecular specificity unmatched by HPLC. Only a few studies have used mass spectrometry to characterize pecan shell bioactive components, despite their high analytical power. Rosa et al. (2011) determined gallic acid and ellagic acid to be the only phenolic compounds in acid hydrolyzed acetonic pecan shell extracts by RPHPLC-ESI-MS [11]. Oligomeric proanthocyanadins were reported to exist in varying degrees of polymerization, from 3 to 10, in shell extracts [23]. In the present study, protonated and deprotonated ions produced using electrospray ionization with a typical fragmentation setting of acid hydrolyzed Nacono pecan shell extracts were monitored simultaneously with ion mode switching every second (Figure 4). Spectral data were digitally processed with the Advion data express software. The background signal was subtracted from the peak ion chromatogram signal to improve the spectral resolution of the mass spectrums.

Bioactive components were identified and characterized by FIA-ESI-MS of acidified methanol (1% HCl) soluble components of pecan shell Nacono ethanol extracts (Table 3). The major components identified were lignin degradation products (lignols, dilignols, trilignols, and oligolignols) and hydrolysis products from other polymeric components. Lignin is the second most abundant biomaterial on the planet and can be found in the secondary layer of plant cell walls. Lignin belongs to a large class of plant secondary metabolites called phenylpropanoids [24]. Structurally, lignin is composed of repeating crosslinked units of lignols. Lignols are categorized according to the degree of oxygen substitution on the phenyl ring. The H-lignols (p-coumaryl alcohol) consist of one hydroxyl group. G-lignols (Coniferyl alcohol) contain one hydroxy and one methoxy group, and S-lignols (Sinapyl alcohol) display one hydroxyl and two methoxy groups. Lignin is often characterized by the ratio of H:G:S subunits [25]. Protonated ions of G(β-O-4′)G fragments at *m*/*z* 195 (phenolic 8-end) and coniferyl alcohol g-structure lignol (aliphatic 4-end) at *m*/*z* 180 are likely products of lignin depolymerization by acidified methanol extraction. Deprotonated guiacylpropane (166 u) at *m*/*z* 165 was formed following the loss of formaldehyde (CH_2_O, 30 u) from the later 8-phenolic end fragment [26]. Fragments of the aliphatic 4-end of G (β-5′)G dilignol were detected in the protonated form at *m*/*z* 222 and in the deprotonated form at *m*/*z* 221.The least abundant fragment of G-structure dilignols observed was protonated phenolic 8-end of the β-β′ resinol linkage at *m*/*z* 206 [27]. Samples rich in different lignin monomer g-subunits indicate a relatively high abundance of g-interunits present in pecan shell extracts [24].

Other monomeric phenolics were identified. Deprotonated vanillyl alcohol (154 u) was detected at *m*/*z* 154 [26]. The most abundant deprotonated component was at *m*/*z* 143. Its molecular structure was not elucidated. The protonated form of sinapyl alcohol, the S-unit lignol, was detected in low abundance at *m*/*z* 211. Bonds associated with s-subunit dilignols are more resistant to cleavage. Low quantities of S-structure lignols may be due to the low temperature and the weak acid hydrolysis extraction conditions used [24,25,26,27,28]. Protonated lignols were also detected at *m*/*z* 116, 143, and 160, deprotonated ion at *m*/*z* 112, and 2-hydroxy-2,4-dienoate at *m*/*z* 112. The only identifiable dilignol was deprotonated guaiacylglycerol-B-guaiacylether dilignol, at *m*/*z* 319 [26,27,28,29]. Proanthocyanidin A was detected in low abundance (peak area 0.5 %) in the positive ion mode at *m*/*z* 593. Mass spectrums of deprotonated ions between 300 and 1200 *m*/*z* showed evidence of highly polymerized components. There was a low abundance of components greater than 500 u detected in the positive ion mode. Various phenylpropanoid derivatives were the main components in ethanolic pecan shell extracts.

## 4. Conclusions

The results from this study indicate that pecan shell by-products have the potential to be used as a natural source of antioxidants in various food applications. The cultivar significantly affected (*p* < 0.05) the total phenolic content and antiradical activity. The extraction method significantly affected (*p* < 0.05) the phenolic content, but not the antiradical activity. Among 20 tested cultivars, the shell extracts from Caddo provided the highest levels of phenolics and antiradical activity (Folin–Ciocalteu and DPPH). The extracts obtained by solid-liquid extraction with ethanol were significantly higher in phenolics, compared to those obtained using distilled water; however, no significant difference was observed in antiradical activity. Crude extracts were extracted with acidified methanol (1% HCL), which resulted in the removal of the interfering material and allowed for the elution of two components in either extract. The first and most abundant peak was attributed to gallic acid, while the other peak did not resemble phenolic standards. The antiradical activity of pecan shell extracts is attributed to a wide variety of bioactive compounds from the class of phenylpropanoids. The major components in ethanolic extracts identified by FIA-ESI-MS were a range of phenylpropanoid derivatives, including phenolic acids, flavonoids, and lignols with varying degrees of polymerization. The significance of these findings has the potential to create new revenue streams for shell by-products, thereby increasing the economic value of the southern United States pecan crop.

## Figures and Tables

**Table 1:** Yield of pecan shell extracts from 20 different cultivars.

Cultivar	Extract Yield	
	mg Dry Extract/g Defatted Shell Powder	
	Aqueous Extracts	Ethanolic Extracts
Desirable	263	25
Caddo	492	281
Elliot	286	175
Nacono	214	305
Oconee	307	176
Pawnee	260	240
Point Coupee	481	144
Curtis	257	66
Kiowa	165	174
Moreland	203	162
Cherokee	351	314
Schley	201	63
Success	145	35
Sumner	222	3
Gloria Grande	221	139
Cape Fear	394	372
Creek	96	273
Maramec	253	50
Jackson	89	265
Melrose	90	65
Average	250	166

**Table 2:** Estimated phenolic content and antioxidant activity of shell extracts.

Cultivar	TPC ^A^		DPPH ^B^	
	(mg GAE g^−1^ Dry Extract)		(mg TE g^−1^ Dry Extract)	
	Aqueous Extracts	Ethanolic Extracts	Aqueous Extracts	Ethanolic Extracts
Pawnee ^C^	202.4 ^ab^	195.5 ^yz^	666.5 ^ab^	608.4 ^wxyz^
Caddo	176.8 ^becd^	212.2 ^xyz^	600.6 ^b^	680.4 ^vwxyz^
Oconee	175.7 ^bcde^	183.3 ^yz^	599.7 ^b^	571.1 ^xyz^
Nacono	174.1 ^bcde^	179.2 ^yz^	574.2 ^b^	580.2 ^xyz^
Desirable	167.0 ^bcde^	209.8 ^xyz^	690.6 ^ab^	611.9 ^wxyz^
Elliot	130.7 ^fe^	234.9 ^xyz^	468.3 ^b^	768.2 ^vwxy^
Curtis ^D^	253.8 ^a^	209.9 ^yz^	934.9 ^a^	820.4 ^vw^
Schley	197.3 ^bc^	194.1 ^yz^	667.4 ^ab^	547.5 ^yz^
Point Coupee	189.5 ^bcd^	304.2 ^x^	612.6 ^b^	796.1 ^vwx^
Sumner	175.6 ^bcde^	195.5 ^yz^	569.6 ^b^	544.3 ^z^
Kiowa	173.3 ^bcde^	190.2 ^xyz^	656.8 ^b^	581.7 ^xyz^
Success	167.45 ^bcde^	173.6 ^yz^	606.3 ^b^	542.5 ^z^
Cherokee	165.2 ^bcdef^	153.5 ^z^	630.2 ^b^	652.9 ^vwxyz^
Moreland	150.4 ^bcdef^	215.4 ^xyz^	718.6 ^ab^	630.8 ^vwxyz^
Maramec ^E^	184.2 ^bcd^	263.2 ^xy^	522.6 ^b^	840.6 ^v^
Gloria Grande	149.8 ^fecd^	231.6 ^xyz^	630.5 ^b^	733.0 ^vwxyz^
Creek	149.5 ^cdef^	202.8 ^yz^	638.7 ^b^	650.5 ^vwxyz^
Cape Fear	143.2 ^def^	203.4 ^yz^	606.4 ^b^	526.7 ^z^
Melrose	126.0 ^ef^	220.3 ^xyz^	495.3 ^b^	668.4 ^vwxyz^
Jackson	114.7 ^f^	227.9 ^xyz^	538.1 ^b^	710.5 ^vwxyz^
Average ± SE	168 ± 6.8	210 ± 7.3	659 ± 21	619 ± 22

^A^ Total extractable phenolic content (Folin–Ciocalteu assay) expressed in mg gallic acid equivalents per gram of free-dried extract. ^B^ Free-radical scavenging activity (DPPH assay) expressed in mg trolox equivalents per gram of freeze-dried extract. ^C^ Demo orchard—2005 est. ^D^ Pathology orchard—1988 est. ^E^ Northwest orchard—1981 est. Values in a column that share a lower-case letter are not significantly different (*p* < 0.05).

**Table 3:** Identified bioactive components characterized by FIA-ESI-MS of acidified methanol (1% HCl) soluble components of pecan shell Nacono ethanol extracts.

*m*/*z*	Compound (MW)	Peak Area	% Peak Area	Maximum Intensity (c/s)
ESI (−)				
112	2-Hydroxypenta-2,4-dienoate UNKNOWN(113 u)	1.5 × 10^6^	3.9	1.2 × 10^6^
154	vanillyl alcohol (154 u)	3.4 × 10^5^	0.9	2.9 × 10^5^
165	guaiacylpropane (166 u)	3.5 × 10^5^	0.9	3.7 × 10^5^
206	phenolic 8-end of G(β-β′)G dilignol	3.6 × 10^5^	0.9	8.0 × 10^5^
221	aliphatic 4-end of G(β-5′)G dilignol	4.2 × 10^5^	1.1	3.7 × 10^5^
248	unknown *	1.6 × 10^6^	4.1	1.5 × 10^6^
319	guaiacylglycerol- β -guaiacylether (320 u)	8.9 × 10^5^	2.3	7.6 × 10^5^
ESI (+)				
116	Unknown *	1.2 × 10^7^	6.3	8.4 × 10^6^
143	Unknown *	2.7 × 10^7^	14.7	2.1 × 10^7^
160	Unknown *	8.1 × 10^5^	0.4	6.1 × 10^5^
180	coniferyl alcohol G-lignol (180 u)	3.7 × 10^6^	2.0	2.4 × 10^6^
195	phenolic 8-end G(β-O-4′)G dilignol	5.2 × 10^6^	2.8	2.4 × 10^6^
211	sinapyl alcohol S-lignol (210 u)	7.8 × 10^5^	0.5	3.8 × 10^5^
222	aliphatic 4-end of G(β-5′)G dilignol	1.9 × 10^7^	10.5	1.5 × 10^7^
593	proanthocyanadin A (593 u)	1.0 × 10^6^	0.5	5.1 × 10^5^

* unknown = not determined.

## References

[1] Statista (2019). Worldwide Sales of Organic Foods 1999–2017 (in Billion U.S. Dollars). https://www.statista.com/statistics/273090/worldwide-sales-of-organic-foods-since-1999/.

[2] United States Department of Agriculture Economic Research Service (ERS) (2017). Organic Market Overview. https://www.ers.usda.gov/topics/natural-resources-environment/organic-agriculture/organic-market-overview.aspx.

[3] United States Department of Agriculture National Agriculture Statistics Service (NASS) (2018). National Statistics for Pecan. https://www.nass.usda.gov/Statistics_by_Subject/result.php?7345CB67-26CC3917AF5BC7828216D668&sector=CROPS&group=FRUIT%20%26%20TREE%20NUTS&comm=PECANS.

[4] Worley R.E. (1994). Pecan Physiology and Composition. Pecan Technology.

[5] Villarreal-Lozoya J.E., Lombardini L., Cisneros-Zevallos L. (2007). Phytochemical constituents and antioxidant capacity of different pecan [Carya illinoinensis (Wangenh.) K. Koch] cultivars. Food Chem..

[6] Prado A.C.P., Silva H.S., Silveira S.M., Barreto P.L.M., Vieira C.R.W., Maraschini M., Ferreira A.R.S., Block J.M. (2014). Effect of the extraction process on the phenolic compounds profile and the antioxidant and antimicrobial activity of extracts of pecan nut [Carya illinoinensis (Wangenh) C. Koch] shell. Ind. Crop. Prod..

[7] Rosa L.A., Vazquez-Flores A.A., Alvarez-Parrilla E., Rodrigo-Garcia J., Medina-Camposc O.N., A’vila-Nava A., Gonza’lez-Reyes S., Pedraza-Chaverri J. (2014). Content of major classes of polyphenolic compounds, antioxidant, antiproliferative, and cell protective activity of pecan crude extracts and their fractions. J. Funct. Foods.

[8] Flores-Córdova M.A., Sánchez Chávez E., Chávez-Mendoza C., García-Hernández J.L., Preciado-Rangel P. (2016). Bioactive compounds and phytonutrients in edible part and nutshell of pecan (*Carya illinoinensis*). Cogent Food Agric..

[9] Huang C.H., Riskowski G.L., Chang J., Lin C.H., Lai J.T., Chang A.C. (2020). Pecan shell by-products—phenolic compound contents and antimicrobial properties. AIMS Agric. Food.

[10] Yemmireddy V.K., Cason C., Moreira J., Adhikari A. (2020). Effect of pecan variety and the method of extraction on the antimicrobial activity of pecan shell extracts against different foodborne pathogens and their efficacy on food matrices. Food Control.

[11] Rosa L.A., Alvarez-Parrilla E., Shahidi F. (2011). Phenolic Compounds and Antioxidant Activity of Kernels and Shells of Mexican Pecan (*Carya illinoinensis*). J. Agric. Food Chem..

[12] Prado A.C.P., Aragão A.M., Fett R., Block J.M. (2009). Antioxidant Properties of Pecan Nut [*Carya illinoinensis* (Wangenh.) C. Koch] Shell Infusion. Grasas Aceites.

[13] Malik N., Perez J.L., Lombardini L., Cornacchia R., Cisneros-Zevallos L., Braford J. (2009). Phenolic compounds and fatty acid composition of organic and conventional grown pecan kernels. J. Sci. Food Agric..

[14] Prado A.C.P., Manion B.A., Seetharaman K., Deschamps F.C., Arellano D.B., Block J.M. (2013). Relationship between antioxdiant properties and chemical composition of the oil and the shell of pecan nuts [*Carya illinoinensis* (Wangenh) C. Koch]. Ind. Crops Prod..

[15] Singleton V.L., Orthofer R., Lamuela-Raventos R.M. (1999). Analysis of Total Phenols and Other Oxidation Substrates and Antioxidants by Means of Folin-Ciocalteu Reagent. Methods Enzymol..

[16] Brand-Williams W., Cuvelier M.E., Berset C. (1995). Use of a free radical method to evaluate antioxidant activity. LWT Food Sci. Technol..

[17] Kureck I., Policarpi P.d., Toaldo I.M., Oliveira M.V., Bordignon-Luiz M.T., Manique P.L., Block J.M. (2018). Chemical Characterization and Release of Polyphenols from Pecan Nut Shell [*Carya illinoinensis* (Wangenh) C. Koch] in Zein Microparticles for Bioactive Applications. Plant Foods Hum. Nutr..

[18] Sánchez-Rangel J.C., Benavides J., Heredia J.B., Cisneros-Zevallos L., Jacobo-Velázquez D.A. (2013). The Folin–Ciocalteu assay revisited: Improvement of its specificity for total phenolic content determination. Anal. Methods.

[19] Chun K.O., Kim D. (2004). Consideration on equivalent chemicals in total phenolic assay of chlorogenic acid-rich plums. Food Res. Int..

[20] Soobrattee M.A., Neergheen V.S., Luximon-Ramma A., Aruoma O.I., Bahorun T. (2005). Phenolics as potential therapeutic agents: Mechanism and actions. Fundam. Mol. Mech. Mutagenesis.

[21] Hagerman A.E. (2002). Tannin Chemistry.

[22] Gosselink R.J.A. (2011). Lignin as a Renewable Aromatic Resource for the Chemical Industry. Ph.D. Thesis.

[23] Vazquez-Flores A.A., Wong-Paz J.E., Lerma-Herrera M.A., Martinez-Gonzalez A.I., Olivas-Aguirre F.J., Aguilar C.N., Wall-Medrano A., Gonzalez-Aguilar G.A., Alvarez-Parrilla E., Rosa L.A. (2017). Proanthocyanidins from the kernel and shell of pecan (*Carya illinoinensis*): Average degree of polymerization and effects on carbohydrate, lipid, and peptide hydrolysis in a simulated human digestive system. J. Funct. Food.

[24] Banoub J., Delmas G., Joly N., Mackenzie G., Cachet N., Benjelloun-Mlayah B., Delmas M. (2015). A critique on the structural analysis of lignins and application of novel tandem mass spectrometric strategies to determine lignin sequencing. J. Mass Spectrom..

[25] Doherty W.O.S., Mousavioun P., Fellows C.M. (2011). Value-adding to cellulosic ethanol: Lignin polymers. Ind. Crop Prod..

[26] Haupert L.J., Owen B.C., Marcum C.L., Jarrell T.M., Pulliam C.J., Amundson L.M., Narra P., Aqueel M.S., Parsell T.H., Abu-Omar M.M. (2012). Characterization of model compounds of processed lignin and the lignome by using atmospheric pressure ionization tandem mass spectrometry. Fuel.

[27] Kiyota E., Mazzafera P., Sawaya A.C. (2012). Analysis of soluble lignin in sugarcane by ultrahigh performance liquid chromatography tandem mass spectrometry with a do-it-yourself oligomer database. Anal. Chem..

[28] Pandey M.P., Kim C.S. (2010). Lignin Depolymerization and Conversion: A Review of Thermochemical Methods. Chem. Eng. Technol..

[29] Kaiser K., Benner R. (2012). Characterization of Lignin by Gas Chromatography and Mass Spectrometry Using a Simplified CuO Oxidation Method. Anal. Chem..

